# Supplementary material for: Curcugreen Treatment Prevented Splenomegaly and Other Peripheral Organ Abnormalities in 3xTg and 5xFAD Mouse Models of Alzheimer’s Disease
Source: Antioxidants (Basel). 2021 Jun 2;10(6):899. doi: 10.3390/antiox10060899 (PMC8229192; doi:10.3390/antiox10060899)
Supplement: Supplementary file 1 [file antioxidants-10-00899-s001.zip › antioxidants-1190420-supplementary.pdf]

Table 1. Name, sources, catalog number of all the chemicals, antibodies and other accessories used for this study.

**Chemicals**	**Sources**	**Catalog no.**	**Address**
Methylcellulose	Sigma	M0512-1KG	St. Louise, MO
Ethyl acetate	Fisher Scientific	E1955K-1	Waltham, MA
Formic acid	Fisher Scientific	A117-50	Waltham, MA
Acetonitrile	Fisher Scientific	A955-212	Waltham, MA
Methanol	Fisher Scientific	A456-4	Waltham, MA
Curcumin	Sigma	78246-100MG	St. Louise, MO
Ammonium acetate	Sigma	A2706-1L	St. Louise, MO
Hematoxylin and eosin stain	ScyTek	HAE-1	Logan, UT
4′,6-diamidino-2-phenylindole	IHC world	IW-1404	San Diego, CA
Propidium-iodide	Sigma	P4864-10ML	St. Louise, MO
TUNEL staining kit	Abcam	ab66110	Cambridge, MA
Depex mounting media	BDH	361254D	Radnor, PA
Anti-fading medium,	Sigma	F4680-25ML	St. Louise, MO
Protease cocktail inhibitors	Sigma	P8340-5ML	St. Louise, MO
Radioimmunoprecipitation assay (RIPA) buffer	ThermoFisher Scientific	VF298280	Waltham, MA
4-20% Tris-glycine gel	ThermoFisher scientific	XP04202BOX	Waltham, MA
Xylene	VWR	89370-094	Radnor, PA
Absolute alcohol	Fisher Scientific	BP2818-4	Waltham, MA
Supersignal West Femto kit	Thermoscientific	VF296347	Waltham, MA
BCA kit	Thermo Scientific	23225	Waltham, MA
PVDF membrane	EMD-Millipore	IPVH00010	Burlington, MA

Table 2: Different antibodies used in this study

**Antibodies**	**Sources**	**Catalog number**	**Address**
Bcl_2	Santa Cruz Biotechnology	sc-7382	Dallas, TX
pAkt	Cell signaling Technology	4060S	Danvers, MA
Caspase-3	Cell signaling Technology	9661S	Danvers, MA
Caspase-6	Cell signaling Technology	9762S	Danvers, MA
IL-10	Santa Cruz Biotechnology	sc-365858	Dallas, TX
IL-1β	Santa Cruz Biotechnology	sc-12742	Dallas, TX
TNF-α	Santa Cruz Biotechnology	sc-52746	Dallas, TX
pTau	Cell Signaling Technology	20194S	Danvers, MA
pGSK-3β	Cell Signaling Technology	5558S	Danvers, MA
GAPDH	Santa Cruz Biotechnology	sc-365062	Dallas, TX

Table 3: Experimental groups and treatment regimen.

Animal groups	Age (m)	Mice used	Treatment groups	Treatment period (m)	For histology	For curcumin measurement
WT+Vehicle	18	6	Vehicle	2	4	3
3xTg+Vehicle	18	9	Vehicle	2	5	0
3xTg+CGR	18	6	CGR (100 mg/kg)	2	5	4
WT+Vehicle	24	9	Vehicle	2	3	3
3xTg+Vehicle	24	8	Vehicle	2		0
3xTg+CGR	24	6	CGR (100 mg/kg)	2		4
WT+Vehicle	18	6	Vehicle	2	3	4
5xFAD	18	6	Vehicle	2	3	0
5xFAD+CGR	18	7	CGR (100 mg/kg)	2	3	4

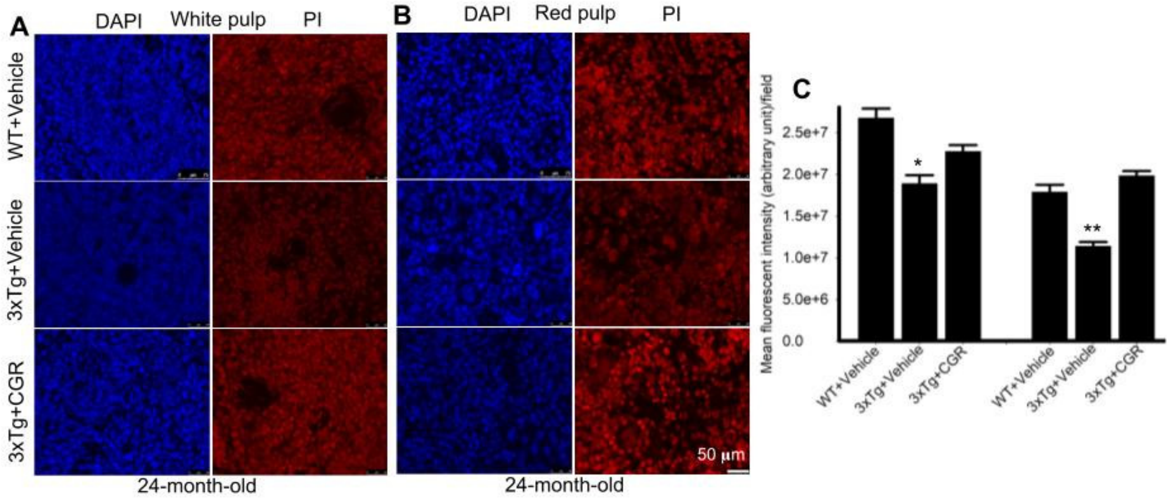

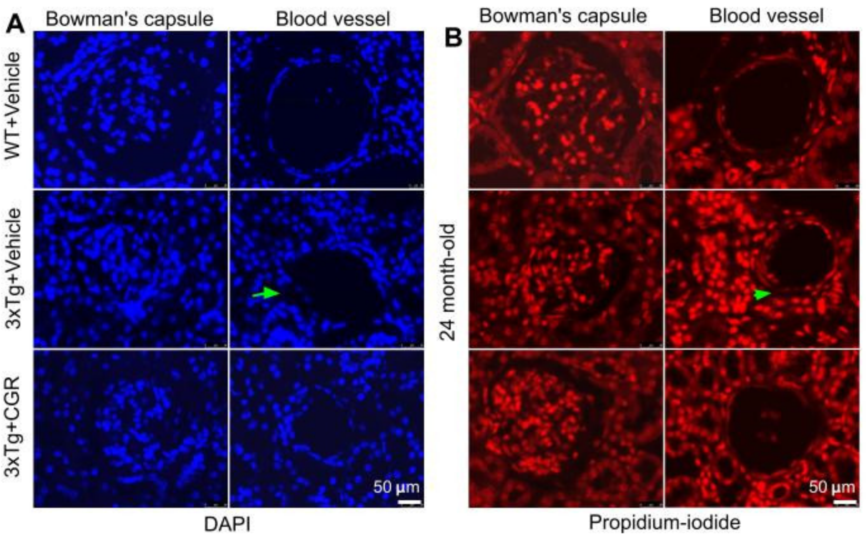
A
Bowman's capsule
Blood vessel
B
Bowman's capsule
Blood vessel
WT+Vehicle
3xTg+Vehicle
3xTg+CGR
24 month-old
50 µm
50 µm
DAPI
Propidium-iodide

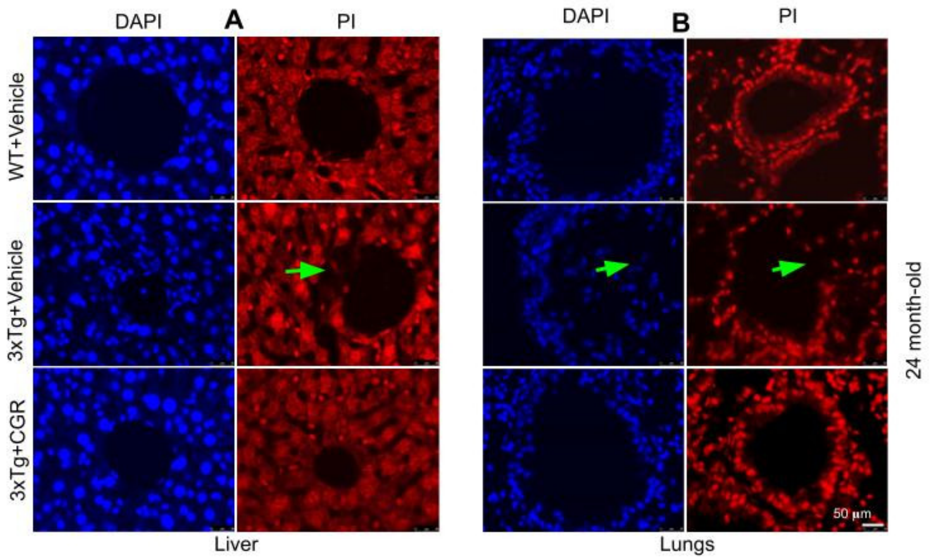
DAPI
A
PI
DAPI
B
PI
WT+Vehicle
3xTg+Vehicle
3xTg+CGR
24 month-old
50 µm
Liver
Lungs
